# Subcellular localisation modulates ubiquitylation and degradation of Ascl1

**DOI:** 10.1038/s41598-018-23056-4

**Published:** 2018-03-15

**Authors:** Sébastien Gillotin, John D. Davies, Anna Philpott

**Affiliations:** 1 Department of Oncology, University of Cambridge, Hutchison/MRC Research Centre, Hills Road, Cambridge, CB2 0XZ UK; 2 Wellcome Trust-Medical Research Council Cambridge Stem Cell Institute, University of Cambridge, Tennis Court Road, Cambridge, CB2 1QR UK

## Abstract

The proneural transcription factor Ascl1 is a master regulator of neurogenesis, coordinating proliferation and differentiation in the central nervous system. While its expression is well characterised, post-translational regulation is much less well understood. Here we demonstrate that a population of chromatin-bound Ascl1 can be found associated with short chains of ubiquitin while cytoplasmic Ascl1 harbours much longer ubiquitin chains. Only cytoplasmic ubiquitylation targets Ascl1 for destruction, which occurs by conjugation of ubiquitin to lysines in the basic helix-loop-helix domain of Ascl1 and requires the E3 ligase Huwe1. In contrast, chromatin-bound Ascl1 associated with short ubiquitin-chains, which can occur on lysines within the N-terminal region or the bHLH domain and is not mediated by Huwe1, is not targeted for ubiquitin-mediated destruction. We therefore offer further insights into post-translational regulation of Ascl1, highlighting complex regulation of ubiquitylation and degradation in the cytoplasm and on chromatin.

## Introduction

During neurogenesis, the fate determination of neural stem cells (NSCs) is coordinated by the activity of proneural basic Helix-Loop-Helix (bHLH) transcription factors and their downstream target genes, acting in a forward genetic cascade during development^1,2^. This hierarchical order of activity also occurs in transcription factor reprogramming models that can directly convert somatic cells into neurons^3,4^. To ensure the correct progression of neuronal differentiation, the expression of proneural transcription factors must be tightly regulated at both the transcriptional and post-translational levels^5–8^.

The proneural factor Ascl1 acts early on in neurogenesis in the central nervous system, particularly in the generation of GABA-ergic interneurons, where it is best characterised as an activator of fate choice and differentiation^9,10^. Recent work demonstrates that it plays an additional role in neural progenitor maintenance during embryonic development, and it also controls cycling of adult neural stem cells between an active state and quiescence^11^. Thus, Ascl1’s central roles in developmental and homeostatic regulation of stem/progenitor proliferation and differentiation have focused attention on the multiple levels of control of this key regulator^12^.

Ascl1 is dynamically regulated in proliferating neural progenitor cells where protein levels oscillate, while commitment to differentiation is accompanied by a shift to sustain Ascl1 expression^13^. In addition, Ascl1 protein is regulated by cdk-dependent multi-site phosphorylation that restrains its ability to drive differentiation in the face of proliferative cues^14^. In common with other bHLH proneural transcription factors, Ascl1 protein has a short half-life and can be targeted for degradation by ubiquitin-mediated proteolysis^7,15^. Recently, Huwe1 (HECT, UBA and WWE domain-containing 1), a HECT domain E3 ubiquitin ligase, has been shown to bind to Ascl1 and target it for proteasome-mediated degradation^11^, a process that is required to allow proliferating adult NSCs to return to quiescence. However, mechanisms and control of Ascl1 ubiquitylation remains to be explored in detail.

Here, we investigate ubiquitin-mediated degradation of Ascl1 during proliferation and differentiation of mammalian neural stem cells (NSCs) and in mouse P19 embryonal carcinoma cells, which represent a classic model system that can respond to proneural protein overexpression by undergoing differentiation^16,17^.

## Results

### Ascl1 degradation rate is similar in proliferating and differentiating neural progenitors

**Figure 1:**
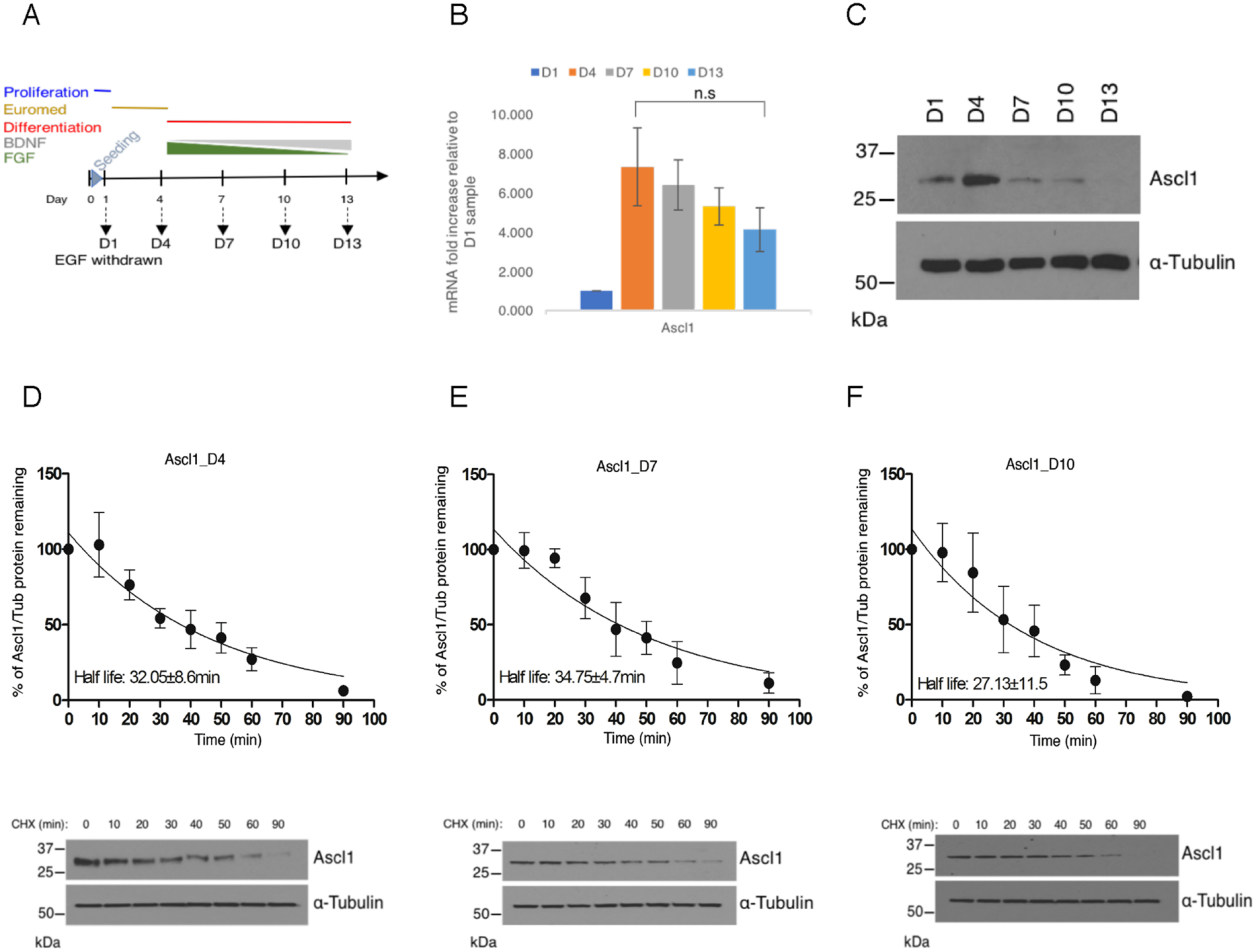
Ascl1 protein is unstable in proliferating and differentiating neural stem cells. (**A**) Schematic representation of the experimental procedure to differentiate neural stem cells and timeline, harvesting time points represented by arrows. (**B**) Endogenous Ascl1 levels measured by qPCR, fold increase is relative to the sample harvested at day 1. Error bars = SEM; n = 3 independent experiments; two-tailed unpaired t-test n.s = p > 0.05. (**C**) Western blot analysis detecting endogenous Ascl1 and α-Tubulin (loading control) in total cell lysate at each time point (cropped); Full length western blots are provided in Supplementary Fig. S1C. (**D**–**F**) Cor3-1 NSCs were treated with cycloheximide at three different time points during neuronal differentiation. Ascl1 protein in whole cell lysates collected at increasing time after cycloheximide addition was detected by western blot and protein abundance quantitated to determine Ascl1 half-life. Representative western blots (cropped) for each time point are shown below the respective graph; Full length western blots for Ascl1 are provided in Supplementary Fig. S1E–G. Error bars = SEM; n = 3 independent experiments.

The stability of a number of proneural transcription factors is tightly regulated to control protein function^7,11^. To determine how the stability of Ascl1 is regulated in proliferating and differentiating NSCs, we used the differentiation protocol established by Spiliotopoulos *et al*.^18^, incorporating slow reduction of FGF with increasing BDNF concentration that promotes enhanced cell viability and a high conversion rate of NS cells to neurons (Fig. 1A). qPCR analysis confirmed that pro-proliferative genes decreased once NSCs were transferred into the differentiation medium (Supplementary Fig. S1A) with a concomitant upregulation of Neurod1 and Tubb3, consistent with neuronal differentiation (Supplementary Fig. S1B). During neuronal differentiation, Ascl1 mRNA reached maximal expression after transfer from the standard proliferation medium to the priming medium (Euromed), where EGF is withdrawn. This was followed by a gradual decline in Ascl1 mRNA levels over the following 9 days of differentiation (Fig. 1B). Following the same time course, we next investigated levels of Ascl1 protein. Maximum expression of Ascl1 protein was reached when cells were transferred to the priming medium (Fig. 1C), and then declined rapidly in differentiation medium to the point where it was undetectable by d13 (9 days in differentiation media). This is consistent with previous studies suggesting that Ascl1 protein levels are tightly regulated in co-ordination with neurogenesis^11,19^. As bHLH transcription factors are known to be regulated at the level of protein half-life, we next examined whether Ascl1 protein stability differs as NSCs progress through the differentiation programme, determining protein abundance after the addition of cycloheximide to block ongoing protein synthesis. In proliferation conditions, Ascl1 half-life was around 30 min as reported previously (^11^ and Supplementary Fig. S1D) and we found that it remained broadly similar throughout the process of differentiation (Fig. 1D–F). Together, these results suggest Ascl1 protein stability is not closely correlated with the transition from proliferation to differentiation, yet a short half-life against a backdrop of declining mRNA contributes to dynamic regulation of Ascl1 protein levels.

### Ascl1 is ubiquitylated differently in the cytoplasm and on chromatin

**Figure 2:**
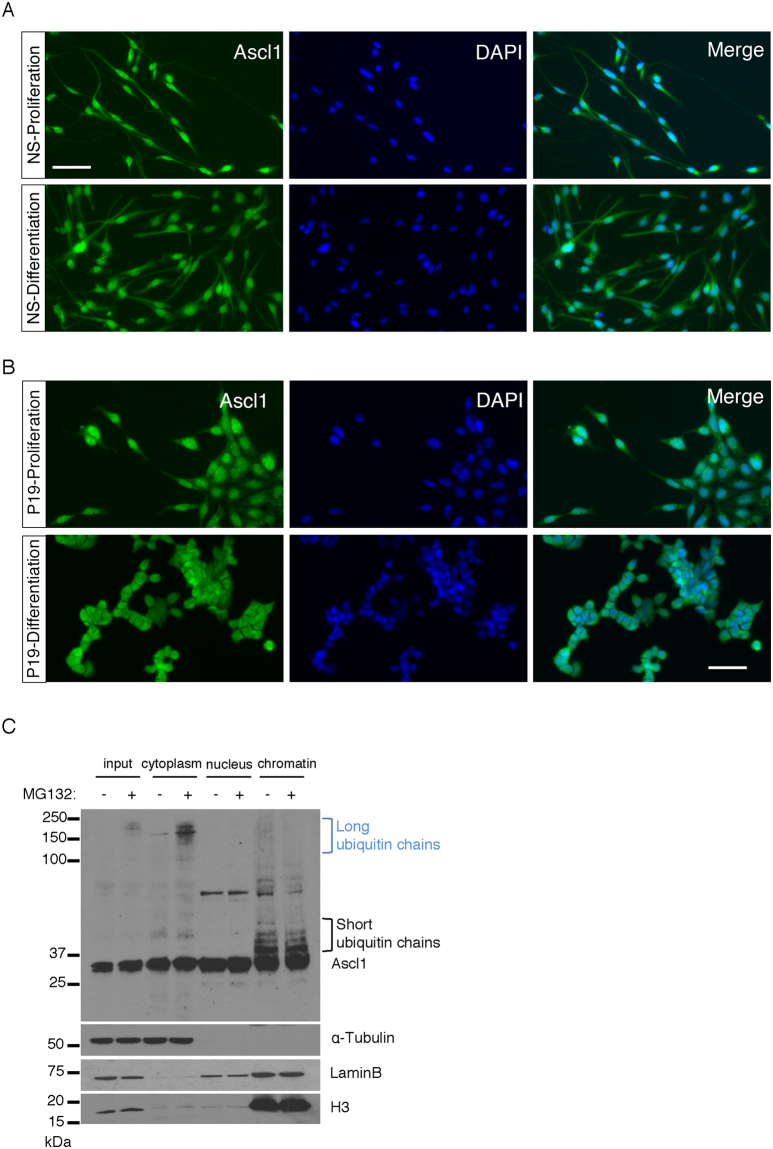
Ascl1 ubiquitylation occurs both in the cytoplasm and on chromatin. (**A**) Immunostaining for Ascl1 (green) and DAPI staining for DNA (blue) in Cor3-1 NSCs under proliferation (day 1; top panels) and differentiation (day 10; bottom panels) conditions. Scale bar, 50 μm. (**B**) P19 cells transfected with (human) Ascl1 (0.25 μg) and immunostained for Ascl1 (green) and DAPI (blue) in proliferation media (top panels) or 24 hours after transfer into differentiation media (bottom panels). Scale bar, 50 μm. (**C**) Neural stem cells at day 4 were either treated with MG132 or DMSO (control) for 2 hours. Cells were fractionated into whole cell lysate (input) and cytoplasm, nucleus and chromatin fractions for western blot analysis. Equal amounts of protein were separated by SDS PAGE and blotted in parallel for endogenous Ascl1, α-Tubulin (loading control for cytoplasmic fraction), LaminB (loading control for nuclear fraction) and histone H3 (loading control for chromatin fraction). Blue bracket, Ascl1 with putative long ubiquitin chains; black bracket, Ascl1 with putative short ubiquitin chains. Full length western blots are provided in Supplementary Fig. S2A and B.

Little is known about the post-translational regulation of Ascl1. Therefore, we next set out to determine mechanisms regulating control of Ascl1 protein, first assessing its sub-cellular localisation. For this, we used two models, looking at endogenous Ascl1 protein in NSCs and looking at overexpressed Ascl1 in mouse P19 embryonic carcinoma cells (Fig. 2A and B respectively). Endogenous Ascl1 protein was detected by immunostaining in both the nucleus and cytoplasm of NSCs with a comparable pattern in proliferation and differentiation media. Similarly, in P19 cells, transfected Ascl1 was detected in both cellular compartments in proliferation and differentiation media. To confirm that Ascl1 could be detected in both subcellular compartments, we performed cellular fractionation of NSCs subject to the NS cells differentiation protocol, fractionating into cytoplasm, nuclear and chromatin components at day 4 in priming medium when Ascl1 protein was at its peak (Fig. 2C).

The fractionation of the cells was efficient, as assayed by α-tubulin, laminB and histone H3 partitioning denoting cytoplasmic, nuclear and chromatin fractions respectively, and Ascl1 protein was detected at a similar level in all 3 sub-cellular compartments. However, strikingly, in addition to monomeric Ascl1, high molecular weight forms of the protein (i.e., >100 kDa, blue bracket) were detected in the cytoplasm fraction. These cytoplasmic slow migrating forms of Ascl1 were enhanced in the presence of the proteasome inhibitor MG132, consistent with their origin being Ascl1 covalently attached to long chains of ubiquitin moieties. In addition, shorter ladders of slower migrating forms of Ascl1 were observed in the chromatin fraction, which were not obviously augmented by the addition of MG132 (black bracket). Similar patterns were observed in cells transferred to differentiation medium (Supplementary Fig. S2C). While these results are consistent with Ascl1 undergoing ubiquitylation in the cytoplasm and also on chromatin, the differing lengths of presumptive ubiquitin chains in the two compartments indicate the possibility of differing regulation.

### Ascl1 is degraded more rapidly in the cytoplasm than on chromatin

**Figure 3:**
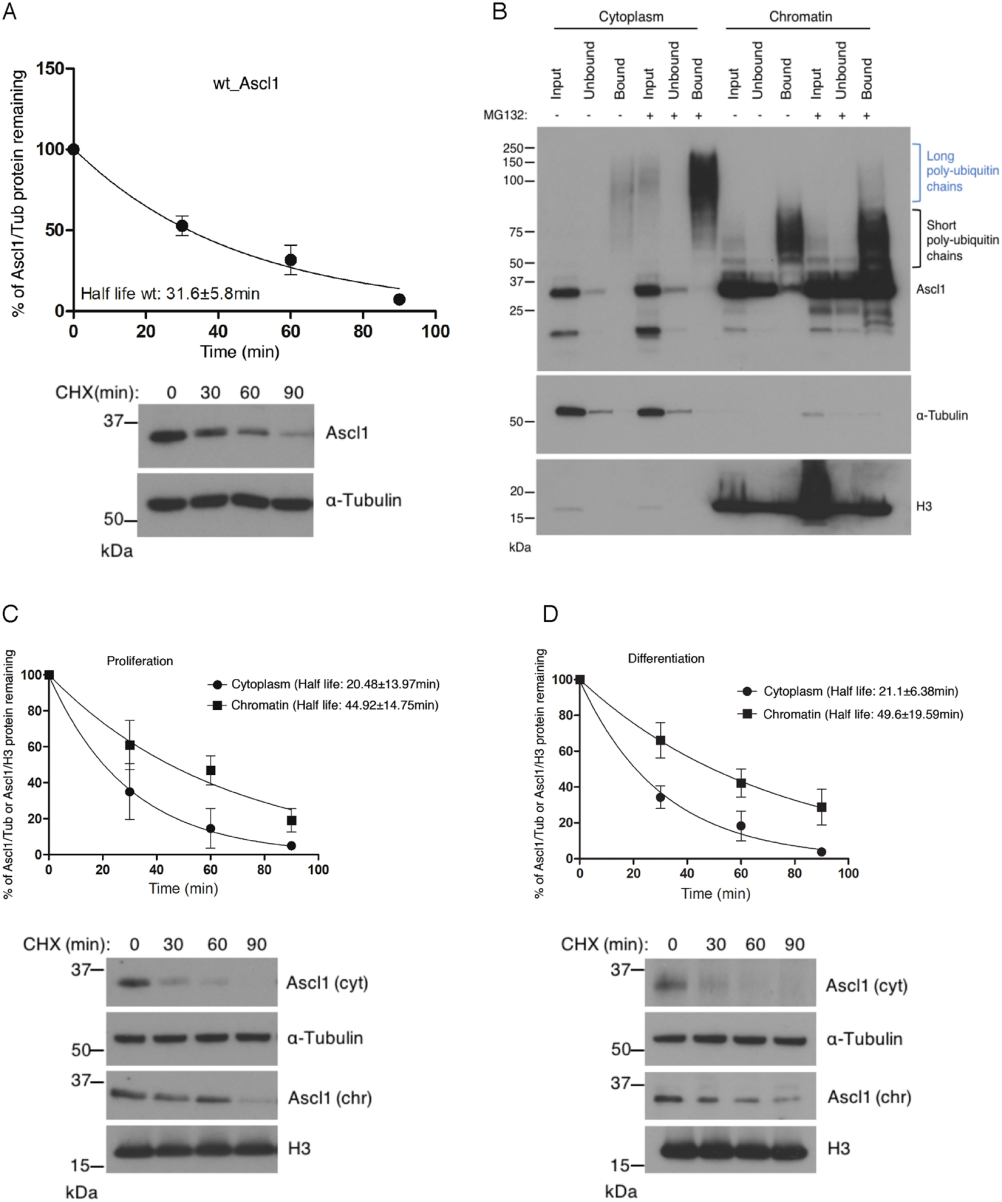
Chromatin-bound Ascl1 is more stable than cytoplasmic Ascl1. (**A**) P19 cells transfected with (human) Ascl1 and growing in proliferation media were treat with cycloheximide to prevent ongoing protein synthesis. Samples were removed at the times indicated and western blot analysis of whole cell lysates was used to determine Ascl1 half-life. Representative western blots (cropped) are shown and full length western blots are provided in Supplementary Fig. S3A. Error bars = SEM; n = 3 independent experiments. (**B**) P19 cells transfected with (human) Ascl1 and transferred the day after to differentiation media for 24 hours. Cellular fractionation was performed after 2 hours treatment with or without MG132 and ubiquitin-bound proteins were isolated using the TUBEs method. Western blotting for Ascl1 compared input, resin-unbound and resin-bound fractions. Blue bracket, Ascl1 with long ubiquitin chains; black bracket, Ascl1 with short ubiquitin chains. Also shown are α-Tubulin (control for cytoplasmic fractionation) and histone H3 (control for chromatin fractionation). (**C**) P19 cells were transfected with (human) Ascl1 and treated the day after with cycloheximide for increasing times before sample withdrawl, as shown. Cytoplasm and chromatin fractions were processed for Ascl1 western blot analysis to determine Ascl1 half-life. Representative western blots (cropped) are shown below and full length western blots are provided in Supplementary Fig. S3B. Error bars = SEM; n = 3 independent experiments. (**D**) As (**C**) but with P19 cells transferred to differentiation media for 24 hours prior to cycloheximide addition. Full length western blots are provided in Supplementary Fig. S3C.

To confirm that ubiquitylation is occurring and then to further characterise the pattern and consequences of Ascl1 ubiquitylation in different cellular compartments, we turned to P19 cells as low levels of endogenous Ascl1 protein in NSCs precluded biochemical analysis. To confirm over-expressed Ascl1 protein is also regulated by ubiquitin-mediated proteolysis in P19 cells in a similar manner to endogenous Ascl1 in NSCs, we first determine Ascl1 protein stability in transfected P19 cells (Fig. 3A). Ascl1 half-life was 31.6 ± 5.8 mins, very similar to the half-life of Ascl1 in NSCs, suggesting the ectopic Ascl1 protein half-life may be regulated in a similar manner to endogenous Ascl1 in NSCs.

To confirm ubiquitylation of transfected Ascl1, we then used the Tandem-repeated Ubiquitin-Binding Entities (TUBEs) method^20^ to directly capture ubiquitylated proteins in cytoplasmic and chromatin fractions (Fig. 3B) and western blotted for Ascl1 to analyse its ubiquitination. Similar to our findings in NSCs, very high molecular weight forms of Ascl1 were captured by the TUBEs from the cytoplasmic fraction of P19 cells confirming that these are indeed ubiquitylated forms of Ascl1 (Fig. 3B, blue bracket). These high molecular weight forms of Ascl1 showed greater accumulation in the presence of MG132, indicating that they were targeting Ascl1 for ubiquitin-mediated degradation. Ubiquitylated forms of Ascl1 were also detected in the chromatin fraction of P19 cells but these ran considerably faster than those seen in the cytoplasmic fraction, indicating the attachment of much shorter ubiquitin chains on chromatin-associated Ascl1 (Fig. 3B, black bracket), as we had observed in NSCs. Addition of MG132 made a more modest difference to accumulation of these forms of Ascl1. To be degraded through the proteasome, the addition of minimum 4 ubiquitin moieties is required^21^, and our results are consistent with short chains of ubiquitin on chromatin-bound Ascl1 being less efficient at driving ubiquitin-mediated proteolysis than the longer chains found in the cytoplasm.

To test whether Ascl1 would have different stability in the two subcellular fractions of P19 cells, we determined the half-life of Ascl1 protein in the cytoplasm and on chromatin after cycloheximide addition in both proliferation and differentiation media (Fig. 3C and D). In the cytoplasm, Ascl1 half-life was around 21 min both in proliferation and in differentiation media but was more than double that in the chromatin fraction under both conditions, consistent with longer ubiquitin chains in the cytoplasm targeting Ascl1 for faster degradation than short chains on chromatin-bound Ascl1.

### Lysines used for ubiquitylation of Ascl1 differ between cytoplasm and chromatin

**Figure 4:**
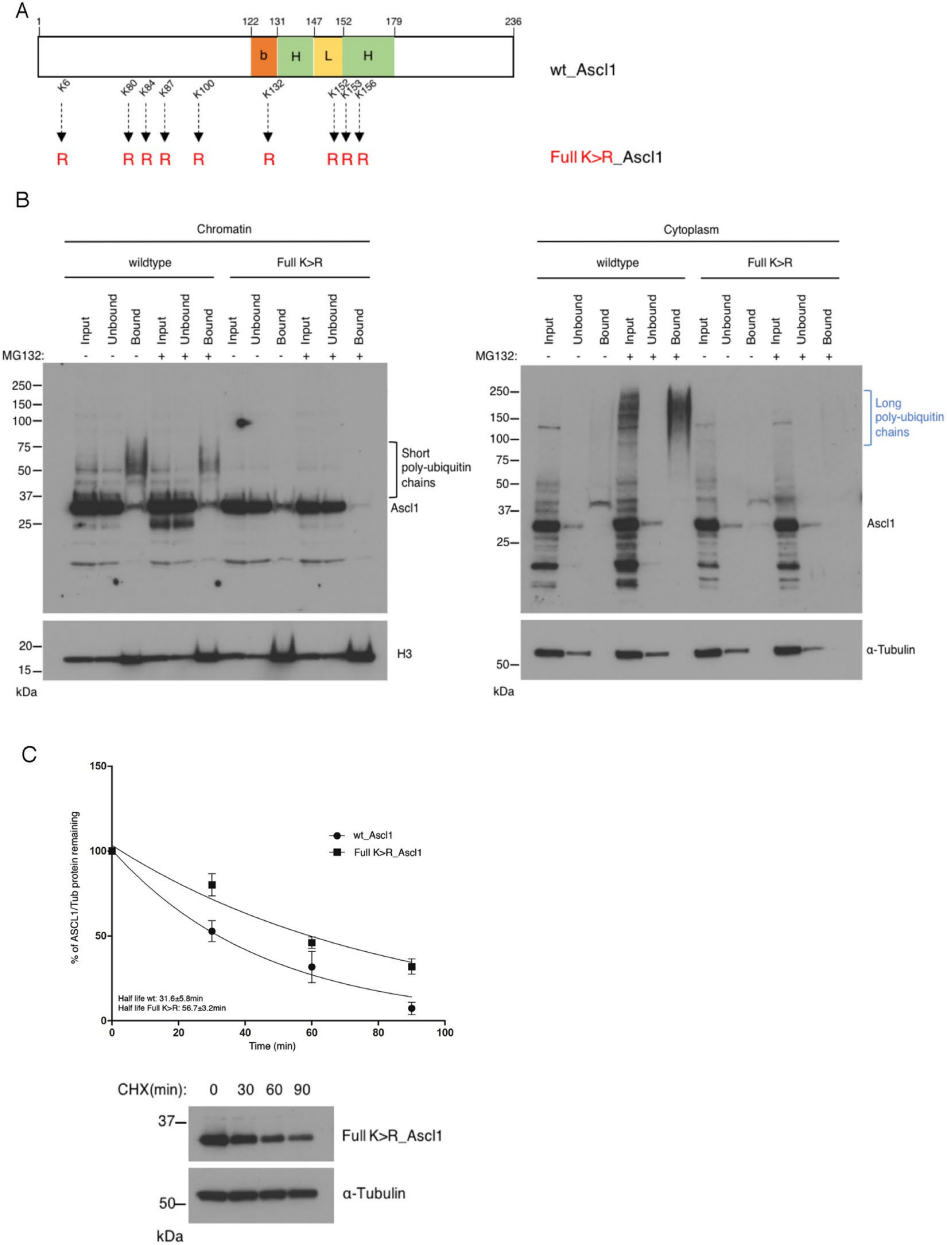
Lysine residues are essential for Ascl1 ubiquitylation. (**A**) Schematic representation of human Ascl1 indicating the localisation of lysine residues that are mutated to arginine in Full K > R_Ascl1. (**B**) P19 cells transfected with (human) Ascl1 and transferred the day after to differentiation media for 24 hours. Cellular fractionation was performed with or without MG132 addition for 2 hours, and ubiquitylated proteins were isolated using the TUBEs method. Western blotting for Ascl1 compared input, resin-unbound and resin-bound fractions from cytoplasmic and chromatin compartments as labelled. Black bracket, Ascl1 with short ubiquitin chains in the chromatin fraction; blue bracket, Ascl1 with long ubiquitin chains in the cytoplasmic fraction. Also shown are α-Tubulin (control for cytoplasmic fractionation) and histone H3 (control for chromatin fractionation). Western blots for loading control were cropped to show specific band of correct molecular weight. (**C**) P19 cells transfected with Full K > R_Ascl1 and growing in proliferation media were treated with cycloheximide for increasing times before sample withdrawl, as shown. Western blot analysis of whole cell lysates was used to determine Full K > R_Ascl1 half-life and compared to wild-type Ascl1 (see Fig. 3A). Representative western blots are shown; Nitrocellulose membrane was cut before probing with the respective primary antibody. Error bars = SEM; n = 3 independent experiments.

Ubiquitylation classically occurs on lysine residues, although ubiquitylation has been reported to occur on the protein N-terminus as well as, highly unusually, on cysteines in the related bHLH proneural factor Neurog2^8^. Sites of ubiquitylation on Ascl1 have not been investigated. Ascl1 has 9 lysine residues in total, with 5 lysines scattered throughout the N-terminus and 4 within the basic helix-loop-helix domains (Fig. 4A). To determine whether ubiquitylation of Ascl1 occurs on canonical lysine residues, we generated a mutant form where all lysines were mutated to arginines (Full K > R_Ascl1, Fig. 4A) and tested its ability to associate with a poly-ubiquitin-binding resin in the TUBEs assay after expression in P19 cells. Mutation of the lysines effectively blocked the formation of the short ladder of ubiquitylated species in the chromatin fraction of Ascl1 even after MG132 addition (Fig. 4B, left panel, black bracket). Similarly, high molecular weight forms of Full K > R_Ascl1 were not identified in the cytoplasm fraction even in the presence of MG132 (Fig. 4B, right panel, blue bracket). This indicates that ubiquitylation of Ascl1 occurs predominantly on canonical lysine residues in both the cytoplasm and on chromatin. Moreover, mutation of all 9 lysines resulted in an almost doubling of Ascl1 half-life from 31.6 ± 5.8 mins to 56.7 ± 3.2 mins, demonstrating that ubiquitylation on lysines contributes to the short half-life of Ascl1, although proteolysis by other mechanisms clearly also limits Ascl1 protein stability.

**Figure 5:**
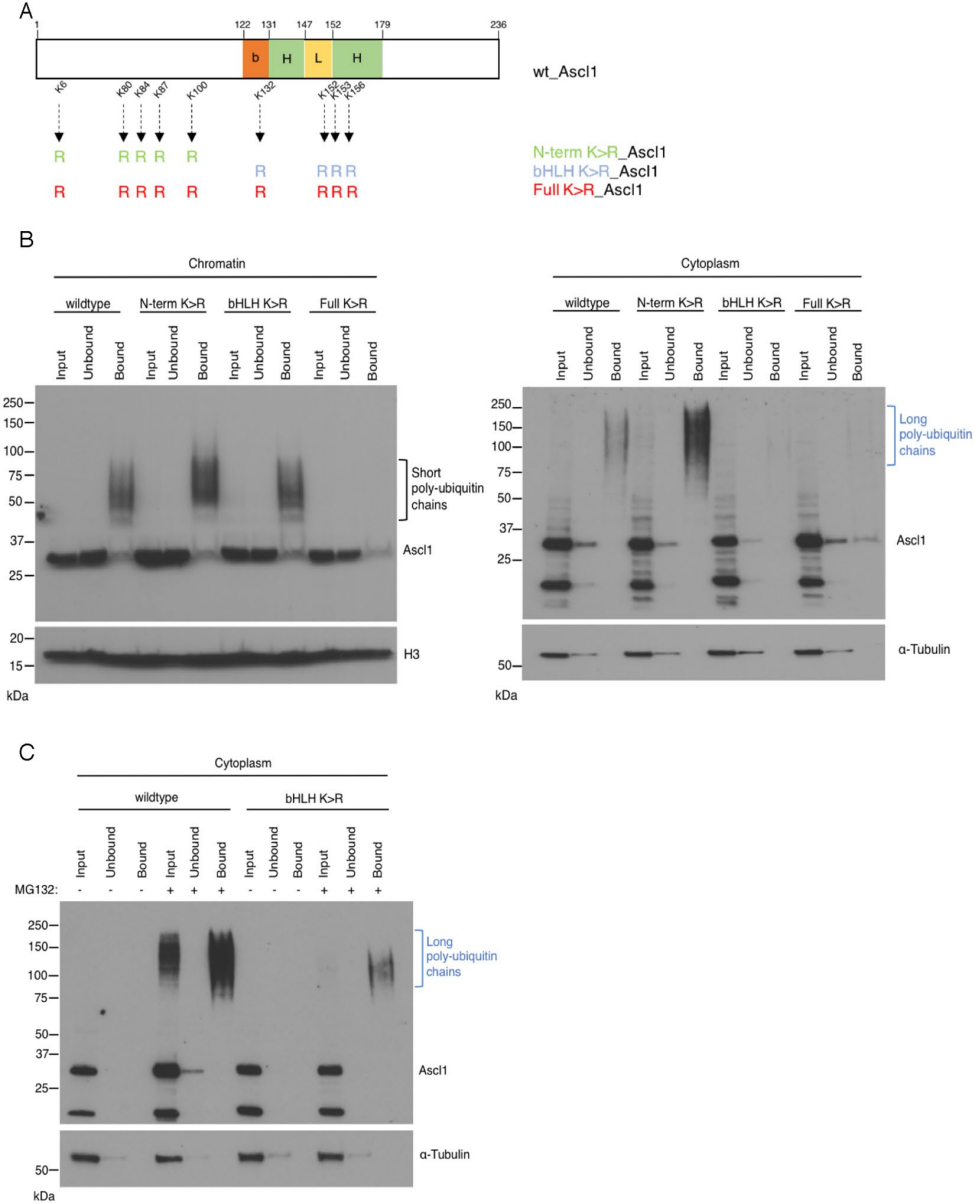
Sites available for ubiquitylation differ in cytoplasmic and chromatin bound Ascl1. (**A**) Schematic representation of human Ascl1 indicating mutation of lysines into arginines at the N-terminus and within the bHLH domain. (**B**) P19 cells transfected with wild-type Ascl1, N-term K > R_Ascl1, bHLH K > R_Ascl1 or Full K > R_Ascl1 were transferred the day after to differentiation media for 24 hours. Cellular fractionation was performed, and ubiquitylated proteins were isolated using the TUBEs method. Western blotting for Ascl1 compared input, resin-unbound and resin-bound fractions from cytoplasmic and chromatin compartments as labelled. Black bracket, Ascl1 with short ubiquitin chains in the chromatin fraction; blue bracket, Ascl1 with long ubiquitin chains in the cytoplasmic fraction. Also shown are α-Tubulin (control for cytoplasmic fractionation) and histone H3 (control for chromatin fractionation). (**C**) As (**B**) above, except cells were incubated with or without MG132 before harvesting to inhibit ubiquitin-mediated proteolysis. Western blots for loading control were cropped to show specific band of correct molecular weight.

While 9 lysines are theoretically available for ubiquitylation, the actual sites of ubiquitin attachment may be influenced by peptide sequence and/or protein structure^22^. Basic helix-loop-helix transcription factors are natively unstructured, but the bHLH region attains structure on dimerization with E proteins and binding to DNA^23^, and this may influence sites of ubiquitylation on chromatin versus cytoplasm. To determine whether sites of Ascl1 ubiquitylation differ in the cytoplasm and on chromatin, we next used the TUBEs assay to pull down forms of ubiquitylated Ascl1 in mutants where lysines had been mutated to arginines either in the N-terminal domain (N-term K > R_Ascl1) or in the bHLH region (bHLH K > R_Ascl1, Fig. 5A). In the chromatin fraction, as before, Ascl1 ubiquitylation was not detected when all lysines were mutated. However, and perhaps surprisingly, on chromatin both the N-term K > R and the bHLH K > R mutants could be ubiquitylated generating conjugates of similar length even without MG132 treatment (Fig. 5B, left panel, black bracket).

In the cytoplasm fraction, we again observed considerably longer ubiquitin chains compared to the chromatin-bound Ascl1 and ubiquitylation was not detected with the Full K > R mutant. However, in contrast to chromatin-bound Ascl1, poly-ubiquitylation occurred on the N-term K > R mutant, and was indeed somewhat enhanced compared to wild-type Ascl1, but the bHLH K > R lysine mutant was not pulled down in absence of MG132, indicating that lysines within the bHLH domain are predominant sites of ubiquitin modification in the cytoplasm (Fig. 5B, right panel, blue bracket). To determine whether any ubiquitylation was possible in the cytoplasm in the absence of bHLH domain lysines, we isolated poly-ubiquitylated forms of wild-type Ascl1 and bHLH K > R_Ascl1 from the cytoplasm fraction using the TUBEs assay (Fig. 5C) in the presence of MG132. In these conditions, wild-type Ascl1 harboured high molecular weights species while the bHLH K > R mutant was weakly detected, indicating that bHLH lysines are the predominant targets of ubiquitylation in the cytoplasm, but that weak ubiquitylation on the N-terminus can be detected when proteolysis is inhibited. Taken together, our data demonstrate that ubiquitylation occurs on a differing pattern of residues in cytoplasmic and chromatin-bound Ascl1.

### Ubiquitylation of chromatin-bound Ascl1 is not required for activity

**Figure 6:**
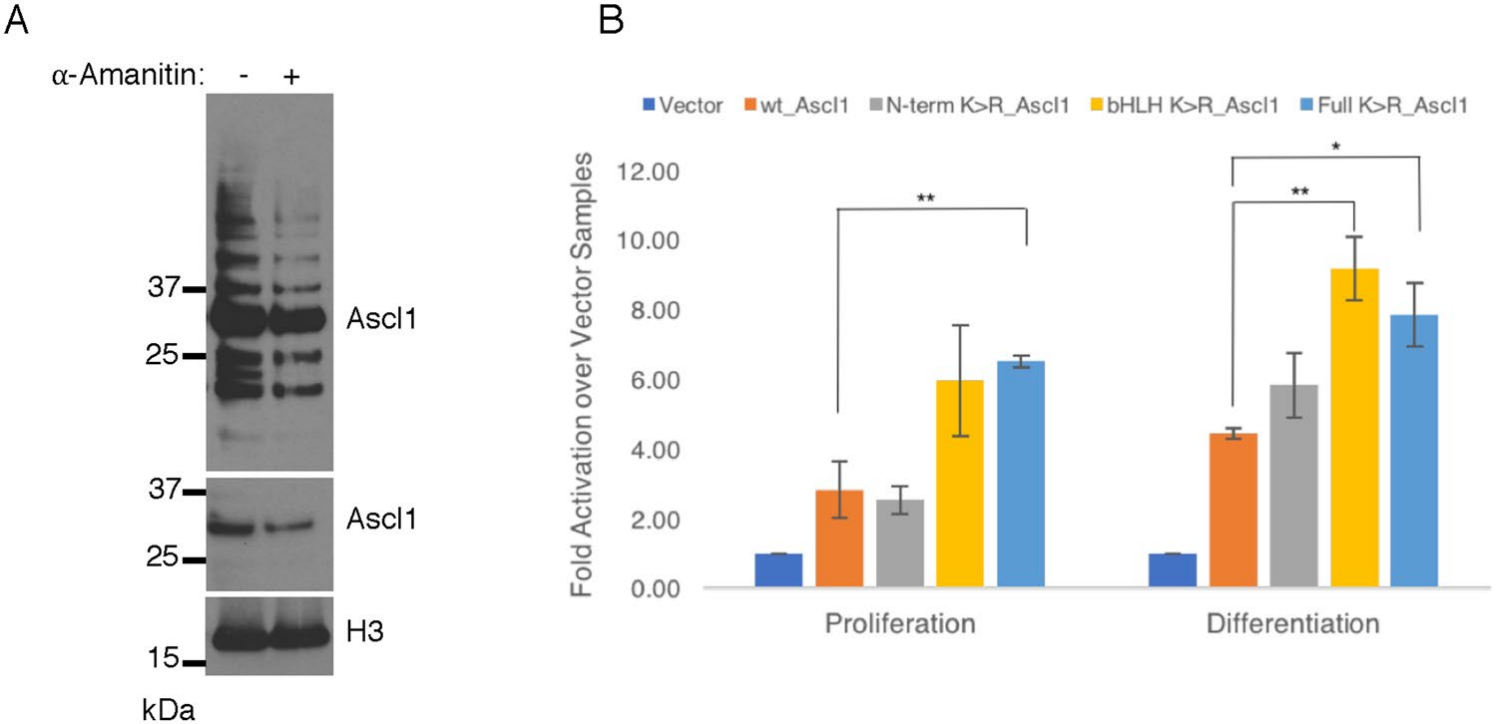
Preventing ubiquitylation of Ascl1 enhances its transcriptional activation. (**A**) P19 cells transfected with wild-type Ascl1 and were transferred the day after to differentiation media for 24 hours, then treated with 20 μg/ml α-Amanitin for 4 hours. Chromatin-bound proteins were isolated and western blotted for Ascl1, top Ascl1 panel long exposure, bottom Ascl1 panel, cropped lower exposure of the same blot to show decrease in Ascl1 protein due to block of ongoing transcription and histone H3 (chromatin marker, cropped to show specific band of correct molecular weight). (**B**) Gene reporter assay in P19 cells transfected with wild-type Ascl1 and mutants thereof as labelled to assay Ascl1 transcriptional activity. Fold activity relative to vector-only controls. Error bars = SEM; n = 3 independent experiments; two-tailed unpaired t-test (*p < 0.05, **p < 0.01).

Ubiquitylation has been previously proposed to play a direct role in the regulation of activity of a variety of transcription factors^24^. For instance, transcriptional activity of the c-Myc protein that also contains a bHLH domain is coupled to its ubiquitylation, and indeed ubiquitin-mediated protein turnover is required to stimulate its transcriptional activity^25^. As short ubiquitin chains are readily detectable on chromatin-bound Ascl1, we investigated whether ubiquitylation of Ascl1 is coupled to its transcriptional activity. For this, we transiently transfected P19 cells with Ascl1 and treated with the transcriptional inhibitor α-Amanitin for 4 hrs in differentiation media before isolation of the chromatin fraction and western blotting for Ascl1 to detect ubiquitylated forms of the protein (Fig. 6A). Acute blocking of transcription using α-Amanitin led to a slight decrease in Ascl1 protein, consistent with a reduction in ongoing transcription of the transfected Ascl1 plasmid. However, Ascl1 ubiquitylation was very similar with and without α-Amanitin addition, indicating that active transcription is not required for ubiquitylation of chromatin-bound Ascl1.

To test whether ubiquitylation of Ascl1 stimulates its transcriptional activity as had been reported for c-Myc^25^ and other transcription factors^24^, we compared the transactivation activity of wild-type Ascl1 with lysine mutants thereof (see Fig. 5A), using a luciferase reporter plasmid containing a part of the Delta promoter previously shown to be bound and activated by Ascl1^26^ (Fig. 6B) in both proliferation and differentiation media. Mutation of all lysines in Ascl1 resulted in almost a doubling of reporter activity, as did mutation of lysines in the bHLH domain, while interestingly mutation of N-terminal lysines had no effect on transcriptional activity. This result indicates that ubiquitylation of Ascl1 on chromatin is not required for transcriptional activity, and indeed preventing ubiquitylation results in a modest increase in activity, consistent with an increase in overall Ascl1 protein levels seen when ubiquitylation is prevented (Fig. 4).

### Huwe1 targets cytoplasmic Ascl1 for degradation

**Figure 7:**
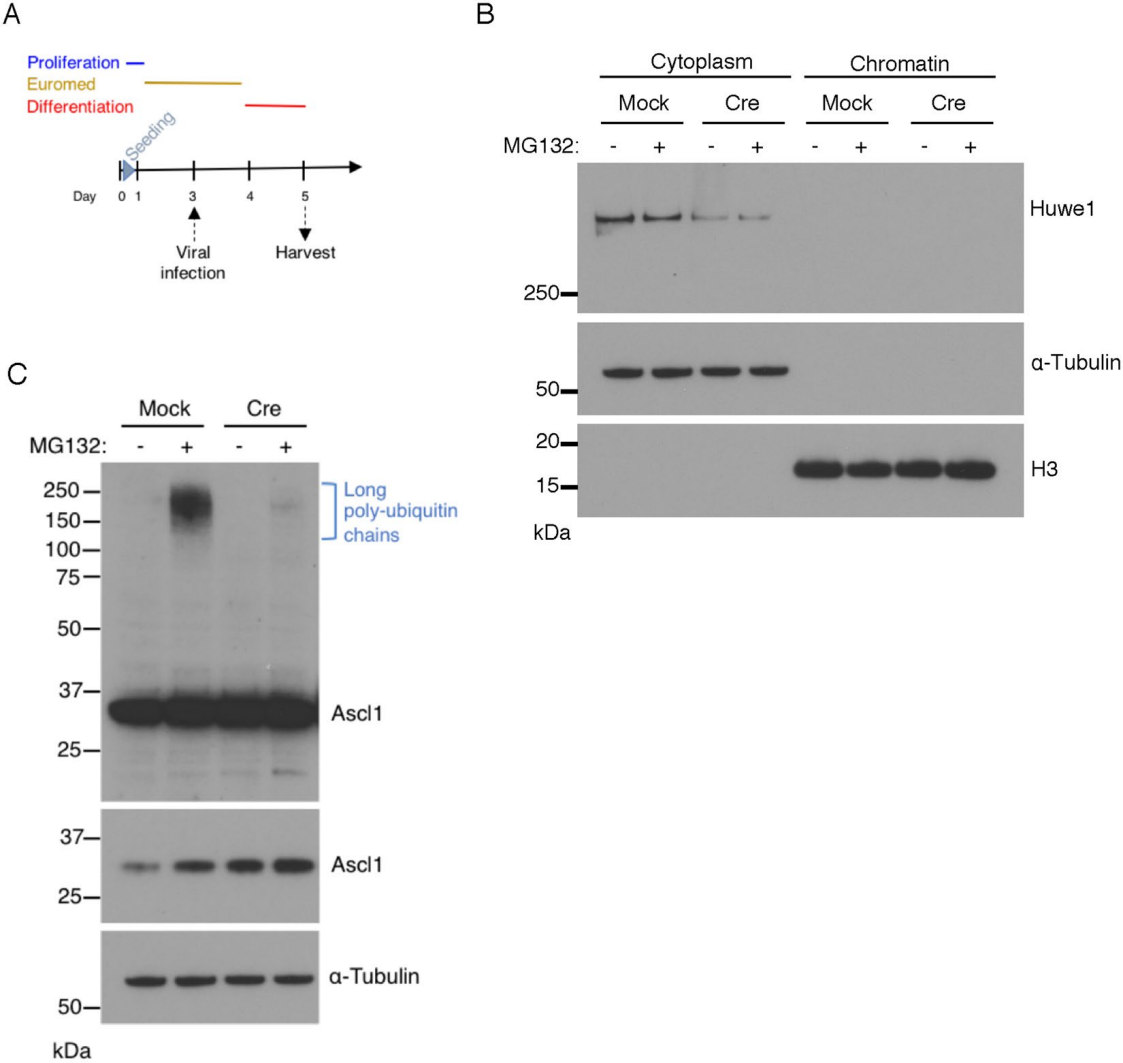
Ascl1 is ubiquitylated by Huwe1 in the cytoplasm. (**A**) Schematic representation of experimental design. Adenovirally-mediated Cre expression was used to delete Huwe1 from NSCs harbouring the Flox-Huwe1 allele and cells were harvested after 1 day in differentiation media. (**B**) Flox-Huwe1 NSCs at day 5 were incubated +/− MG132 for 2 hours, and cytoplasmic and chromatin fractions were processed for western blot analysis to detect Huwe1 as well as α-Tubulin (loading control for cytoplasmic fraction) and histone H3 (loading control for chromatin fraction). (**C**) Cytoplasmic fractions with and without cre-mediated knockdown of Huwe1 (see panel B), were incubated with and without MG132 for 2 hours and western blotted to detect free and ubiquitin-conjugated (blue bracket) forms of Ascl1. Top panel is long exposure to reveal high molecular weight ubiquitylated forms of Ascl1 (blue bracket), bottom Ascl1 panel is lower exposure (cropped from the same blot) to show stabilisation of Ascl1 after Huwe1 knockdown, α-Tubulin (loading control for cytoplasmic fraction, cropped to show specific band of correct molecular weight), n = 3.

Short ubiquitin chains are associated with chromatin-bound Ascl1, but much longer chains are found attached to Ascl1 in the cytoplasm. Longer ubiquitin chains are known to target proteins for degradation more efficiently so we next explored how Ascl1 might be ubiquitylated in the cytoplasmic compartment^27^. The HECT domain ubiquitin ligase Huwe1 is the only E3 ubiquitin ligase currently known to target Ascl1 for ubiquitylation and degradation and knocking out Huwe1 results in an increase in Ascl1 half-life in NSCs^11^. The interaction between Ascl1 and Huwe1 is not dependent on the presence of DNA^11^ and Huwe1 has also been previously detected in the cytoplasm^28,29^. To determine whether Ascl1 is specifically targeted for ubiquitylation by Huwe1 in the cytoplasm we used NSCs that have been engineered to conditionally delete Huwe1 on expression of cre recombinase^11^ (Fig. 7A). Lentivirally-mediated Cre expression resulted in a significant knock-down of Huwe1 in the cytoplasm of a pool of infected cells, as detected by western blotting (Fig. 7B). Huwe1 was not detected on chromatin either before or after knock-out consistent with cytoplasmic localisation reported previously^28,29^.

To determine whether Huwe1 is responsible for the long chains of ubiquitin conjugated to Ascl1 in the cytoplasm, we examined the ubiquitin pattern of Ascl1 in Flox-Huwe1 NSCs after cellular fractionation and western blot analysis (Fig. 7C). High molecular weight of poly-ubiquitylated forms of Ascl1 were detected with the mock viral infection in the presence of MG132 but poly-ubiquitylated Ascl1 forms were severely reduced when Huwe1 was knocked down. On the chromatin, we could not detect a change in the short ubiquitylation ladder associated with Ascl1 but Huwe1 knock-down led to the stabilisation of Ascl1 protein (Supplementary Fig. S4A). Thus, ubiquitylation of Ascl1 in the cytoplasm is predominantly mediated by Huwe1.

All together, these results suggest that Huwe1 targets Ascl1 for ubiquitylation degradation specifically in the cytoplasm, and that preventing cytoplasmic destruction leads to more Ascl1 protein that is available to bind to chromatin.

## Discussion

Ascl1 is a central regulator of neural stem and progenitor maintenance that is dynamically regulated at the protein level^1,11,13^ (Fig. 1). In this study, we explored regulation of ubiquitylation and degradation of Ascl1 in both neural stem cells and in P19 embryonal carcinoma cells that have long served as a model for studying proneural protein-mediated neuronal differentiation^16–18,30,31^. Total cellular Ascl1 has a short half-life of about 30 minutes in both cell lines (Figs 1 and 3), consistent with previous analysis of Ascl1^11^, and with the short half-lives reported for other bHLH proneural proteins^7,8^. It is of note that Ascl1 had a similar half-life in proliferation and at different stages of our differentiation protocol (Fig. 1D,E and F and Supplementary Fig. S1D), indicating that altering the half-life of Ascl1 is not a central mechanism used to regulate proliferation versus differentiation of these cell types. However, our analyses revealed different rates of degradation of Ascl1 in the cytoplasm and when bound to chromatin; chromatin-bound Ascl1 is twice as stable as cytoplasmic Ascl1.

To understand better how degradation of Ascl1 is regulated we looked further at the ubiquitylation of Ascl1 protein in both cytoplasm and on chromatin and uncovered distinct modes of ubiquitin modification in the two sub-cellular compartments. Cytoplasmic Ascl1 is conjugated to long chains of ubiquitin moieties (Fig. 2C), resulting in a substantial decrease in migration of conjugated Ascl1 on SDS PAGE and clear association with a ubiquitin-binding resin (Figs 3B, 4B and 5B). Moreover, addition of the proteasome inhibitor MG132 resulted in significant stabilisation of ubiquitylated Ascl1, demonstrating that these long chains target Ascl1 for proteolysis (Figs 2C, 3B and 4B). In contrast, chromatin-bound Ascl1 displays much shorter and generally less prominent ubiquitin chains (Figs 2C and 3B); for instance chains of only 3 ubiquitin moieties were visible in wild-type Ascl1 P19 cells (Fig. 4B, first lane). Ubiquitin-binding resin concentrates forms of chromatin-bound Ascl1, but even these enriched forms of ubiquitylated Ascl1 run much more rapidly than Ascl1 conjugated to longer chains in the cytoplasm (e.g., Fig. 3B).

We do not see a significant stabilisation of chromatin-bound Ascl1 in the presence of MG132, indicating that ubiquitylation does not target for significant proteasome-mediated destruction in this context (Fig. 3B). Our results are consistent with a requirement for long ubiquitin chains to target proteins to the proteasome^22^ and indeed, we see that chromatin-bound Ascl1 has more than twice the half-life of cytoplasmic Ascl1 (Fig. 3C). Nevertheless, chromatin-bound Ascl1 is still unstable, with a half-life of less than 1 hour, indicating that other modes of Ascl1 destruction contribute to protein turnover. Consistent with this, even in whole cell lysates, Ascl1 mutants lacking all lysines that act as sites for canonical ubiquitylation still readily degraded, albeit less rapidly than the wild-type protein.

Interestingly, ubiquitylation of Ascl1 on the chromatin can occur on either N-terminal or bHLH domain lysines, despite binding to DNA and dimerization with E protein partners^23^, while mutation of bHLH lysines dramatically reduces ubiquitylation in the cytoplasm (Fig. 5). Thus, not only do chromatin-bound and cytoplasmic Ascl1 accumulate ubiquitin chains of differing length, they also differ in the lysines that can be used to anchor those chains.

Ubiquitylation of a number of transcription factors has been described to either stimulate or inhibit their transcriptional activity, with or without activity of the proteasome^24^. In addition, coupling of transcription to ubiquitylation on chromatin has also been reported^25^. We blocked transcription with α-Amanitin and saw no significant effect on Ascl1 ubiquitylation (Fig. 6A), indicating that close coupling between ongoing transcription and Ascl1 modification does not occur. However, we did see a modest but significant increase in the activation of an Ascl1-responsive reporter construct when ubiquitylation was blocked by mutation of lysines (Fig. 6B). Taken together, these results suggest that Ascl1 does not require ubiquitylation for its activity and indeed, that blocking its ubiquitylation enhances transcriptional activity, likely through an increase in protein levels due to a doubling of its half-life (Fig. 4C).

Finally, only the E3 ligase Huwe1 has been identified thus far as targeting Ascl1 for ubiquitylation, though the sub-cellular location where ubiquitylation occurs has not been described. We see that Huwe1 is found exclusively in our cytoplasmic fractions in neural stem cells, and significantly, is not found associated with chromatin so is unlikely to be directly controlling ubiquitination and destruction of Ascl1 at sites of active transcription (Fig. 7). Instead, our results support a model where Huwe1 is acting as the major E3 ligase for Ascl1 but it is targeting it for destruction by ubiquitylation in the cytoplasm. While Huwe1 targets Ascl1 for cytoplasmic destruction and is not present on chromatin, nevertheless, Huwe1 knock-down leads to more chromatin-bound Ascl1 (Supplementary Fig. S4A), presumably because more stable protein shuttles from the cytoplasm to the nucleus. Consistent with this, mutation of lysines in the bHLH domain only inhibit ubiquitylation in the cytoplasm, yet results in enhanced activation of an Ascl1 reporter, again pointing to cytoplasmic stabilisation ultimately resulting in an accumulation of chromatin-bound Ascl1 and leading to an increase in its transcriptional activity.

Taken together, our results point to a more complex regulation of Ascl1 subcellular localisation, stability and activity than has previously been described. As we uncover more about the pivotal role Ascl1 plays in developmental and adult neurogenesis as well as in cellular reprogramming, it will be important to have a complete understanding of regulation of post-translational control of this key master regulator.

## Methods

### Cell culture, neuronal differentiation and cell transfection

Neural stem cells, Cor3–1 were grown in Complete media (DMEM/HAM’S-F12 (Sigma, D8437), 1.45% Glucose (Sigma, G28644), 1 × MEM-NEAA (Gibco, 11140-035), 100 units/ml penicillin and 100 μg/ml streptomycin (Gibco, 15140-122), 0.16% BSA (Gibco, 15260-037), 0.1 mM β-Mercapto-ethanol (Gibco 31350-010), 1% B-27 supplement (Gibco, 17504-044) and 0.5% N2 supplement (Gibco, 17502-048)). Before changing the medium or passaging the cells, 10 ng/ml mouse EGF (Peprotech, 315-09), 10 ng/ml human FGF (R&D systems, 233-FB) and 1 μg/ml Laminin (Sigma, L2020-1MG) were added to the required volume of Complete media. For neuronal differentiation, 24 hours after seeding, cells were transferred into a priming medium Euromed-N (Euroclone) supplemented with 1% B-27 without vitamin A (Gibco, 12587010), 0.5% N2 and 10 ng/ml human FGF for 3 days. Cells were then transferred into a differentiation medium (1:3 ratio DMEM/HAM’S-F12:Neurobasal (Gibco, 21103-049) supplemented with 1% B-27 without vitamin A, 0.5% N2, 10 ng/ml human FGF, 20 ng/ml BDNF (R&D systems, 248-BD) and 1μg/ml Laminin for 3 days followed by a change of medium every 3 days with reduction of the FGF concentration by half to zero and a constant concentration of BDNF at 30 ng/ml.

Neural stem cells (Floxed-Huwe1-RYFP) used for Huwe1 knock-out were described in King *et al*.^32^ and in Urban *et al*., 2017^11^ and maintained as described above. Transduction of CRE recombinase was performed with the adenovirus CMV-CRE (Vector Biolabs, 1045 N) at a MOI of 100. Empty adenovirus (CMV-null) was used as control at the same MOI.

P19 cells were maintained in MEM-α (Gibco, 41061-029) supplemented with 10% FBS and 100 units/ml penicillin and 100 μg/ml streptomycin. For differentiation, cells were transferred into Neurobasal medium supplemented with penicillin/streptomycin, 1% B-27 without vitamin A, 1 × GlutaMAX (Thermo, 35050061) and 1μg/ml Laminin. Differentiation medium was changed every 24 hours if required. P19 cells were transfected at 80% confluence with Lipofectamine 2000 (Invitrogen, 11668-027) in proliferation medium following the manufacturer’s instructions with a 1:2 ratio (DNA:Lipofectamine). A list of plasmids is provided in Table S2 in supplementary information.

### Quantitative Real-Time PCR

Total RNA was extracted from cells using the RNeasy kit (Qiagen). Template cDNAs were synthesised using the QuantiTect Reverse Transcription Kit (Qiagen), and qRT-PCR was performed using QuantiFast SYBR Green Master Mix (Qiagen) in a Lightcycler 480 PCR system (Roche). EiF1α was used as housekeeping gene to normalise each qPCR target and the values were calculated as fold increase to proliferation samples. Thermal cycling conditions: 95 °C for 5 mins, then 40 cycles of 95 °C for 10 seconds, 60 °C for 10 seconds and 72 °C for 30 seconds. The primer sequences are provided in Table S1 in supplementary information. Data are presented as means ± SEM of normalised values from at least three independent experiments.

### Western blotting

Western blots were either performed from total cell lysates obtained by lysing cells directly with RIPA buffer (Sigma-Aldrich) complemented with protease inhibitor cocktail (cOmplete, Roche) or from fractionated lysates (see below). Proteins concentration was determined using BCA protein assay kit (Thermo). SDS-PAGE electrophoresis was carried out in 10% pre-cast polyacrylamide gels (Novex) in MOPS buffer. After transfer onto nitrocellulose, membranes were blocked for 1hr with 10% non-fat milk in TBS-0.1% Tween solution and probed overnight with primary antibody: Ascl1 (1:300, hybridoma supernatant, gift from David Anderson and François Guillemot), Histone H3 (1:10,000, Abcam, ab1791) and α-Tubulin (1:10,000, Abcam, ab7291), LaminB (1:500, Santa Cruz, sc-6216) and Huwe1 (1:1,000, Bethyl Laboratories, A300-486A). Mouse secondary (goat anti-mouse HRP 1:10,000, Millipore, 71045-3) or rabbit secondary (1:5,000, Rabbit IgG HRP linked, GE Healthcare, NA934V) antibodies were used for detection in 5% milk in TBS-0.1% Tween solution for 1 hour. Membranes were developed using ECL Prime (GE Healthcare, RPN2232) for Ascl1 or ECL detection reagents (GE Healthcare, RPN2106) for the other antibodies. All western blots were performed at least in triplicate.

### Cellular Fractionation

Cor3–1 cells were seeded at 1.5 × 10^6^ in 10 cm dishes and P19 were seeded at 1.0 × 10^6^ in 10 cm dishes. P19 were transfected with 3 μg of Ascl1 plasmid constructs (Supplementary Table S2). Cells were washed once with room temperature PBS and scraped into 1 ml PBS. Cells were pelleted at 1,200 rpm in an eppendorf tube at 4 °C for 3 min. Pellets were covered with 5 volumes of ice-cold E1 buffer (50 mM Hepes-KOH pH 7.5, 140 mM NaCl, 1 mM EDTA pH8.0, 10% glycerol, 0.5% NP-40, 0.25% triton X-100, 1 mM DTT) complemented with 1 × protease inhibitor cocktail and were gently pipetted up and down (5 times) before centrifugation at 3,400 rpm at 4 °C for 2 min. The supernatant was collected in a fresh tube (cytoplasm fraction) and the pellet was resuspended by gentle pipetting in the same volume of E1 buffer and centrifuged as before. The supernatant was discarded and the pellet was resuspended by gentle pipetting in the same volume of E1 and left for 10 min on ice. After centrifugation at 3,400 rpm at 4 °C for 2 min, the supernatant was discarded and the pellet was resuspended by gentle pipetting in 2 volumes of ice-cold E2 buffer (10 mM Tris-HCl pH8.0, 200 mM NaCl, 1 mM EDTA pH8.0, 0.5 mM EGTA pH8.0) complemented with 1 × protease inhibitor cocktail and centrifuged at 3,400 rpm at 4 °C for 2 min. The supernatant was collected in a fresh tube (nucleus fraction) and the pellet was resupsended in the same volume of E2 and centrifuged as before. The supernatant was discarded and the pellet was resuspended in the same volume of E2 and left for 10 min on ice. After centrifugation at 3,400 rpm at 4 °C for 2 min, the supernatant was discarded and the pellet was resuspended in ice-cold E3 buffer (500 mM Tris-HCl, 500 mM NaCl) complemented with 1 × protease inhibitor cocktail by gentle pipetting in the same volume used for E1. The solution (chromatin fraction) was sonicated in a Bioruptor (Diagenode) for 5 min on setting H, 30 sec ON/30 sec OFF for western blot analysis or was digested with Benzonase (1:1,000, Novagen, 70664-3) for TUBEs pull down. All fractions were centrifuged at 13,000 rpm at 4 °C for 10 min and protein concentration was determined using BCA protein assay kit.

### Agarose-TUBEs pull down

15 μl of 50% Agarose-TUBEs (LifeSensors, UM401) were used to pull down between 350 μg and 450 μg of ubiquitylated proteins from the cytoplasm and chromatin fractions in 500 μl final volume of respective buffers. Incubation was performed for 1 hour at 4 °C on a rocker platform. Agarose-TUBEs were collected by centrifugation at 1,000 × g at 4 °C for 5 min. The supernatant was collected in a fresh tube (unbound fraction). Agarose-TUBEs were washed 3 times following the manufacturer’s instructions and were boiled in reducing sample buffer (25 μl) for 5 min, centrifuged at maximum speed in a top bench centrifuge for 1 min and eluted samples were loaded onto gels (Bound fraction: 15 μl for Ascl1 and 5 μl for loading controls) alongside 5 μg of proteins from the unbound fraction and 5 μg from fractionated lysates (Input).

### Protein Stability Assay

Cor3-1 cells or transfected P19 cells (2 μg Ascl1 plasmid DNA in 6 cm dishes) were treated with 10 μg/ml cycloheximide (Sigma-Aldrich, C4859). At define time points, cells were harvested with RIPA lysis buffer (Sigma-Aldrich, R0278) or fractioned. Equal amount of each whole cell lysate or fractionated lysate was separated on SDS-PAGE and immmunoblotted with mouse anti-Ascl1 and mouse anti-α-Tubulin (1:10,000, Abcam). Protein levels were quantified with FiJi software. Half-lives were calculated using a nonlinear regression analysis (One phase decay, Prism).

### Immunocytochemistry

Cor3-1 or P19 cells were plated onto ECM-coated (Sigma, E1270) 24-well plate (25,000 cells per well). At indicated time, cells were fixed in 4% paraformaldehyde for 10 min at room temperature. Cells were then washed twice in PBS and permeabilised in 0.2% Triton X-100 PBS for 10 min at room temperature. Blocking was performed in PBS with 3% BSA for 60 min at room temperature. Anti-Ascl1 primary antibody (Abcam, ab74065) was used at 5 μg/ml final concentration in PBS with 2% FBS and 0.02% Triton X-100 overnight at 4 °C without rocking. The next day, cells were washed 3 times in 0.02% Triton X-100 PBS for 5 mins and incubate with secondary antibody (Alexa488 goat anti-rabbit IgG, Life Technologies) diluted 1:500 in PBS with 2% FBS and 0.02% Triton X-100 for 1 hour at room temperature in the dark. Cells were next washed 3 times in 0.02% Triton X-100 PBS for 5 mins and incubated in 4′,6-diamidino-2-phenylindole (DAPI, 1:10,000) in PBS for 20 min at room temperature in the dark. After 2 washes in PBS, fluorescence labelling was visualised with Axio Scan (Zeiss).

### Gene Reporter Assay

P19 cells were plated into 48-well plates (75,000 cells per well) and transfected the day after with 100 ng ΔM short-Luciferase (Supplementary Table S2), 50 ng pRL-TK (Renilla, Promega) and 100 ng Ascl1 using Lipofectamine 2000 in proliferation media. One set of cells was harvested the day after (proliferation samples) with the Dual-Luciferase Reporter Assay System (Promega, E1910) while the other set of cells was transferred into differentiation media and harvested 24 hours later. Luciferase assay was performed following the manufacturer’s instructions (Promega) using a Tecan Infinite 200Pro microplate reader. Values for Luciferase were normalised to Renilla-Luciferase values and data are presented as means ± SEM of duplicate and experiments were repeated three times.

### Data availability

All data generated or analysed during this study are included in this published article (and its Supplementary Information files).

## Electronic supplementary material

Supplementary Information
