# Supplementary material for: Subcellular localisation modulates ubiquitylation and degradation of Ascl1
Source: Sci Rep. 2018 Mar 15;8:4625. doi: 10.1038/s41598-018-23056-4 (PMC5854709; doi:10.1038/s41598-018-23056-4)
Supplement: Supplementary file 1 — Supplementary Information [file 41598_2018_23056_MOESM1_ESM.pdf]

Supplementary Information

Subcellular localisation modulates ubiquitylation and degradation of Ascl1

Sébastien Gillotin[1,2], John D Davies[1,2] and Anna Philpott[1,2]

[1] Department of Oncology, University of Cambridge, Hutchison/MRC Research Centre, Hills Road, Cambridge CB2 0XZ, UK

[2] Wellcome Trust-Medical Research Council Cambridge Stem Cell Institute, University of Cambridge, Tennis Court Road, Cambridge CB2 1QR, UK

Supplementary Figure S1:

(A) qRT-PCR analysis of mRNA expression levels of pro-proliferative marker genes (Cdk1, Cdk2, E2f1 and Skp2) in Cor3-1 NSCs total mRNA. Fold increase is relative to the sample harvested at day 1. Error bars= SEM; n=3 independent experiments.
(B) qRT-PCR analysis of mRNA expression levels of neuronal differentiation genes (Neurod1 and Tubb3) in Cor3-1 NSCs total mRNA. Fold increase is relative to the sample harvested at day 1. Error bars= SEM; n=3 independent experiments.
(C) Full length gels for Fig. 1C. Nitrocellulose membrane was cut before probing with the respective primary antibody.
(D) Cor3-1 NSCs were treated with cycloheximide in proliferation medium and processed for western blot analysis of whole cell lysates to determine Ascl1 half-life. Representative western blot is shown below the graph. Error bars= SEM; n=3 independent experiments.
(E) Ascl1 Full length gels for Fig. 1D
(F) Ascl1 Full length gels for Fig. 1E
(G) Ascl1 Full length gels for Fig. 1F

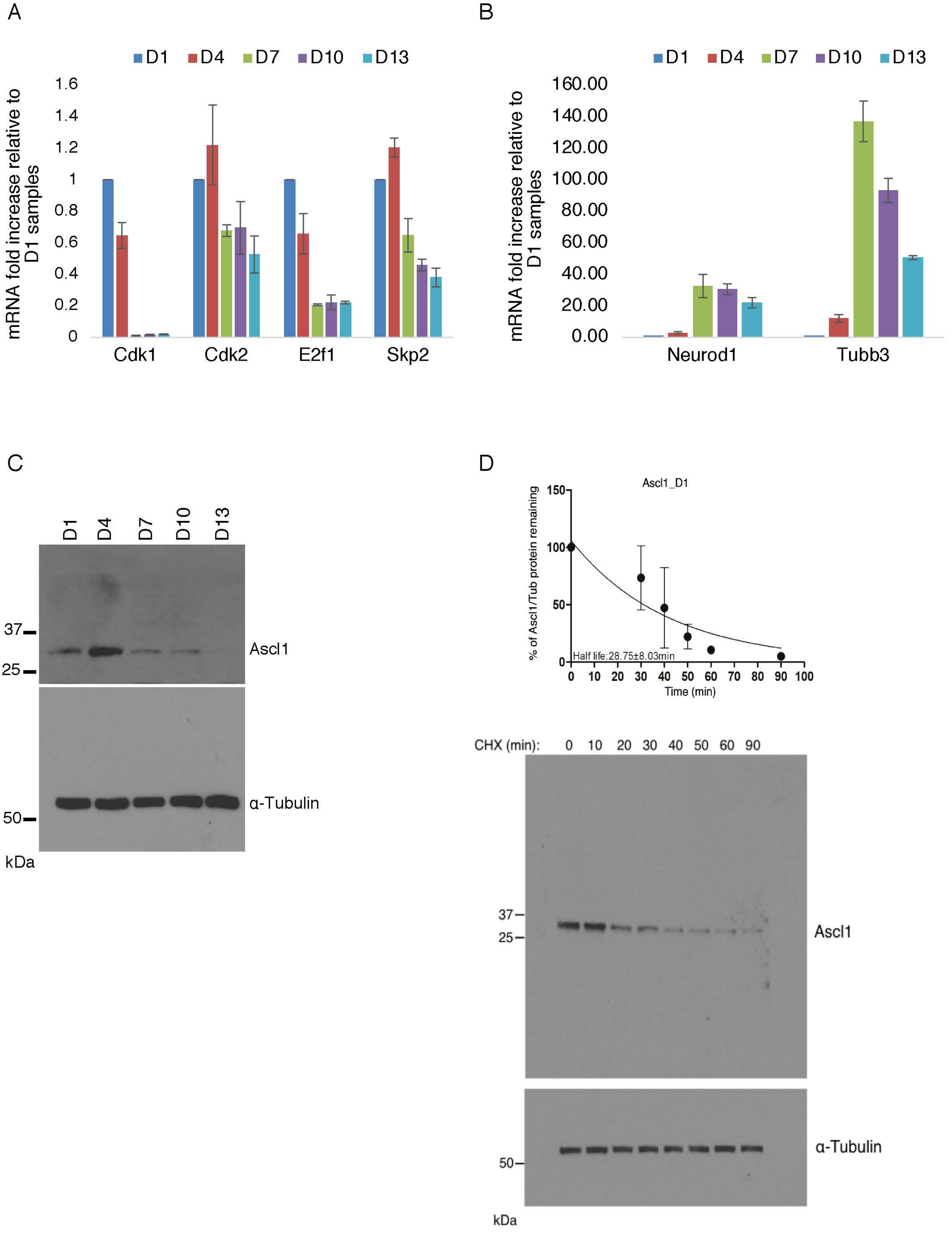
A
D1 D4 D7 D10 D13
mRNA fold increase relative to D1 samples
1.6
1.4
1.2
1
0.8
0.6
0.4
0.2
0
Cdk1
Cdk2
E2f1
Skp2
B
D1 D4 D7 D10 D13
mRNA fold increase relative to D1 samples
160.00
140.00
120.00
100.00
80.00
60.00
40.00
20.00
0.00
Neurod1
Tubb3
C
D1 D4 D7 D10 D13
37
25
Ascl1
α-Tubulin
50
kDa
D
Ascl1_D1
% of Ascl1/Tub protein remaining
150
100
50
0
Half life:28.75±8.03min
0 10 20 30 40 50 60 70 80 90 100
Time (min)
CHX (min): 0 10 20 30 40 50 60 90
37
25
Ascl1
α-Tubulin
50
kDa

E

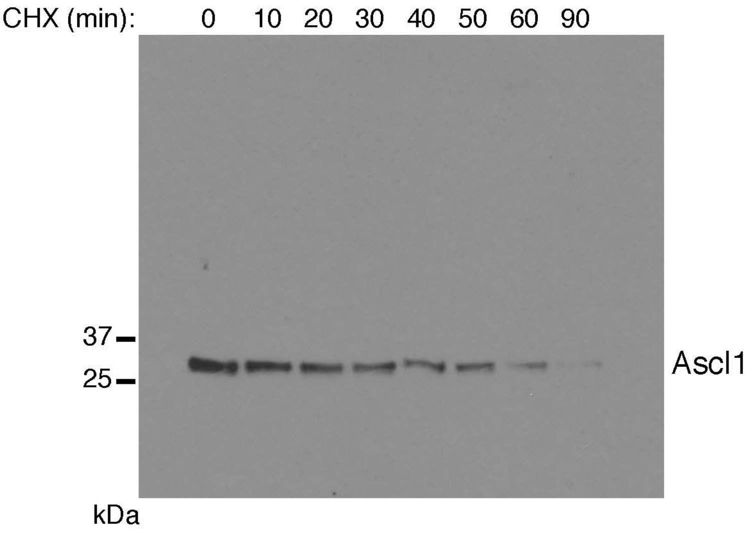
CHX (min): 0 10 20 30 40 50 60 90
37
25
kDa
Ascl1

F

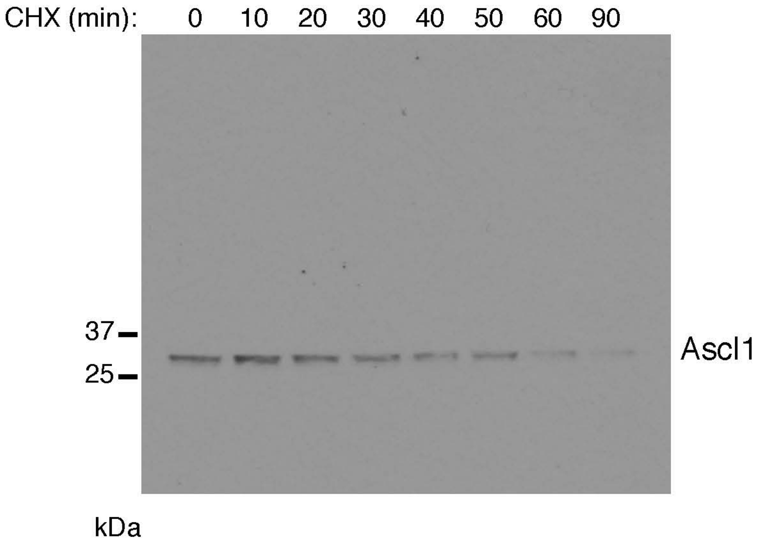
CHX (min): 0 10 20 30 40 50 60 90
37
25
kDa
Ascl1

G

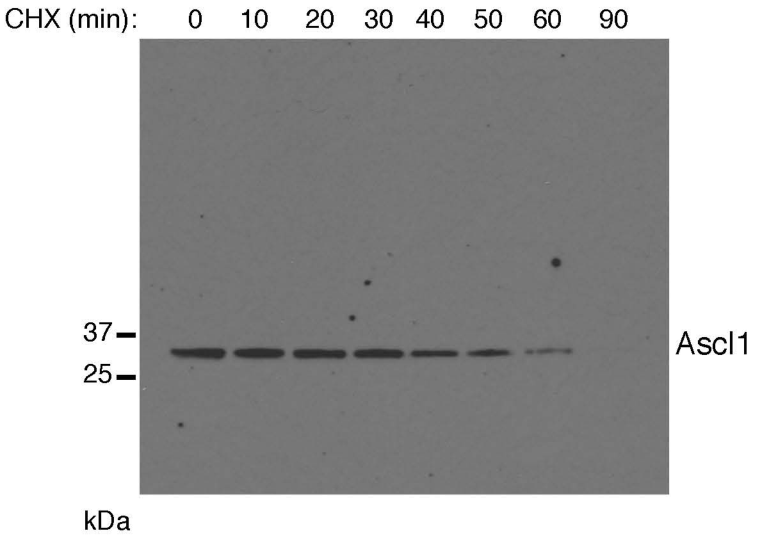
CHX (min): 0 10 20 30 40 50 60 90
37
25
kDa
Ascl1

Supplementary Figure S2:

(A) Full length western blot for α-Tubulin (loading control for cytoplasmic fraction) for Fig. 2C.
(B) Full length western blot for LaminB (loading control for nuclear fraction) and histone H3 (loading control for chromatin fraction) for Fig. 2C. Nitrocellulose membrane was cut before probing with the respective primary antibody.
(C) Cor3-1 NSCs at day 5 of the neuronal differentiation protocol were either treated with MG132 or DMSO (control) for 2 hours. Cells were fractionated into whole cell lysate (input) and cytoplasm, nucleus and chromatin fractions for western blot analysis. Equal amounts of protein were separated by SDS PAGE and blotted for endogenous Ascl1, α-Tubulin (loading control for cytoplasmic fraction), LaminB (loading control for nuclear fraction) and histone H3 (loading control for chromatin fraction). Blue bracket, Ascl1 with long ubiquitin chains; black bracket, Ascl1 with short ubiquitin chains.

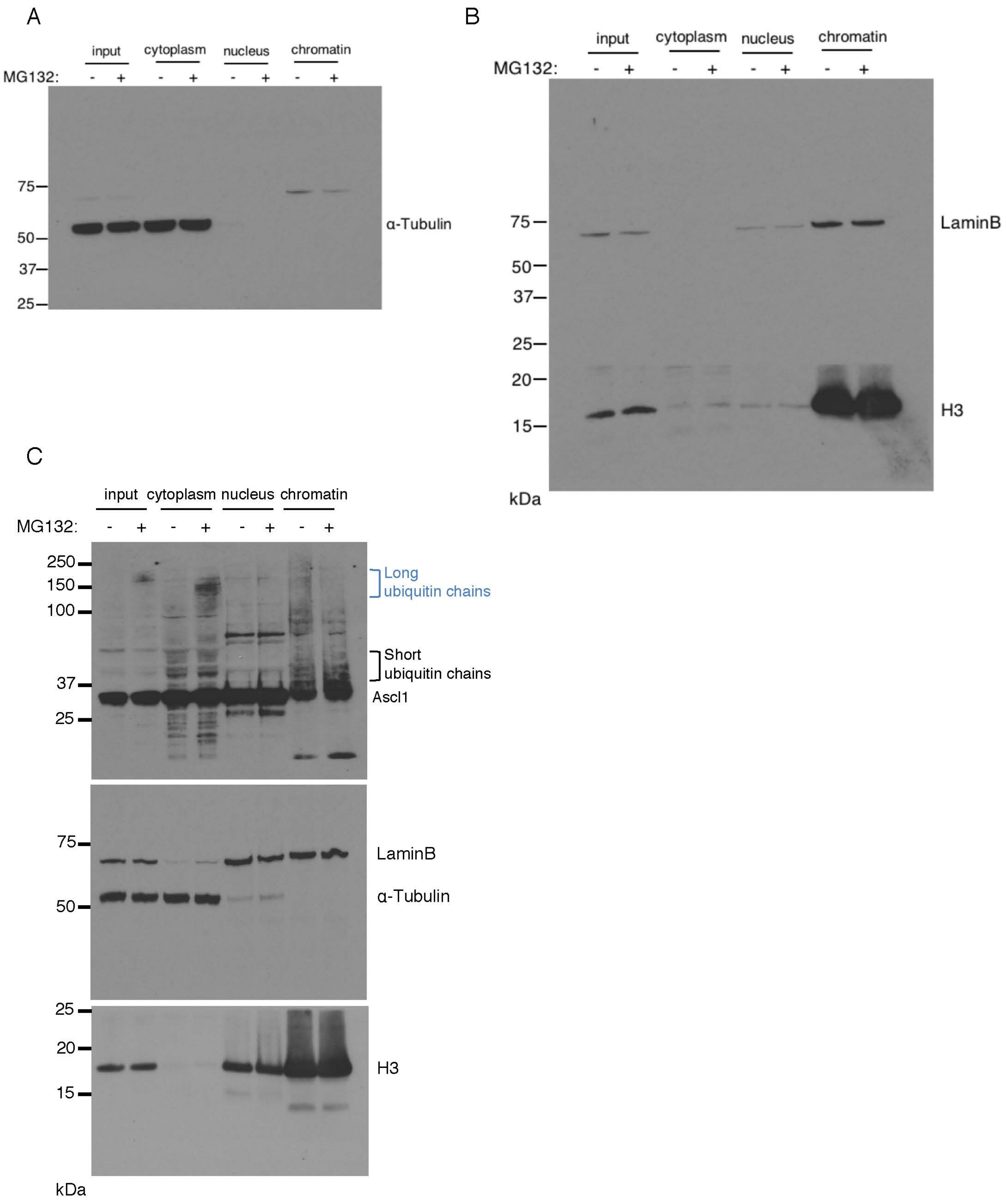
A
input
cytoplasm
nucleus
chromatin
MG132: - + - + - + - +
75
50
37
25
α-Tubulin
B
input
cytoplasm
nucleus
chromatin
MG132: - + - + - + - +
75
50
37
25
20
15
LaminB
H3
kDa
C
input cytoplasm nucleus chromatin
MG132: - + - + - + - +
250
150
100
37
25
Long ubiquitin chains
Short ubiquitin chains
Ascl1
75
50
LaminB
α-Tubulin
25
20
15
H3
kDa

Supplementary Figure S3:

(A) Full length western blots for Ascl1 (top panel) and for α-Tubulin (bottom panel; loading control for cytoplasmic fraction) for Fig. 3A.
(B) Full length western blots for Ascl1 (top panel; cytoplasmic fraction), α-Tubulin (second panel; loading control for cytoplasmic fraction), Ascl1 (third panel; chromatin fraction) and histone H3 (bottom panel; loading control for chromatin fraction) for Fig. 3C. Nitrocellulose membrane for each fraction was cut before probing with the respective primary antibody.
(C) Full length western blots for Ascl1 (top panel; cytoplasmic fraction), α-Tubulin (second panel; loading control for cytoplasmic fraction), Ascl1 (third panel; chromatin fraction) and histone H3 (bottom panel; loading control for chromatin fraction) for Fig. 3D. Nitrocellulose membrane for each fraction was cut before probing with the respective primary antibody.

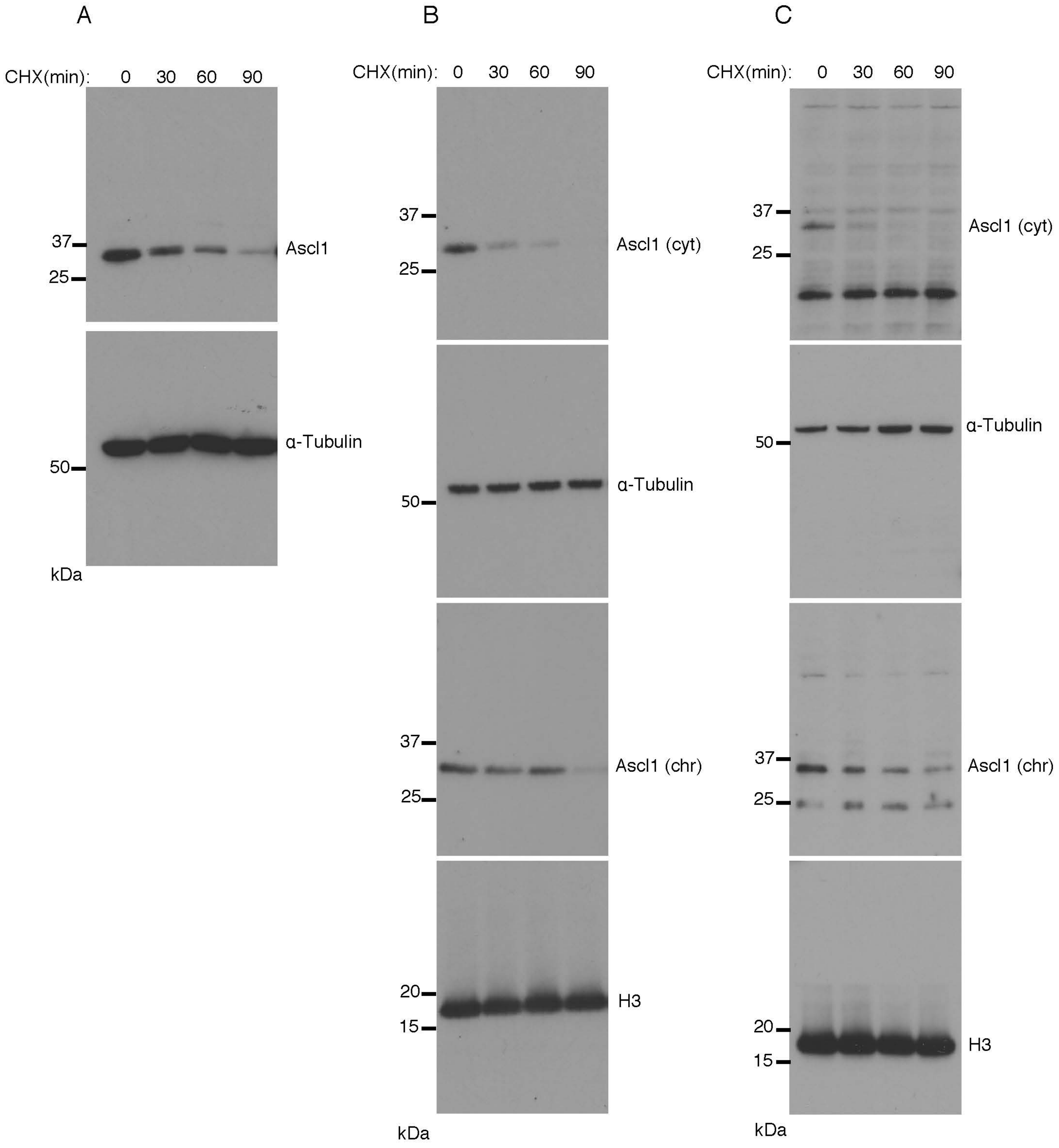
A
CHX(min): 0 30 60 90
37
25
Ascl1
50
α-Tubulin
kDa
B
CHX(min): 0 30 60 90
37
25
Ascl1 (cyt)
50
α-Tubulin
37
25
Ascl1 (chr)
20
15
H3
kDa
C
CHX(min): 0 30 60 90
37
25
Ascl1 (cyt)
50
α-Tubulin
37
25
Ascl1 (chr)
20
15
H3
kDa

Supplementary Figure S4

(A) Chromatin fractions, with and without cre-mediated knockdown of Huwe1 were incubated with and without MG132 for 2 hours and western blotted to detect free and ubiquitin-conjugated (black bracket) forms of Ascl1. Top panel is long exposure to reveal short molecular weight ubiquitylated forms of Ascl1 (black bracket), bottom Ascl1 panel is short exposure (cropped from the same blot) to show stabilisation of Ascl1 after Huwe1 knockdown, histone H3 (loading control for chromatin fraction, cropped to show specific band of correct molecular weight), n=3.

A

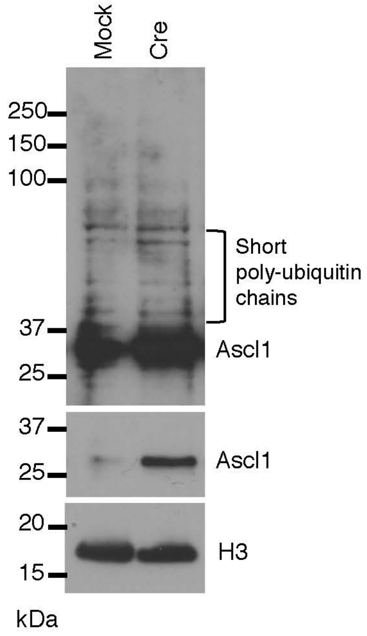
Mock
Cre
250
150
100
37
25
Short
poly-ubiquitin
chains
Ascl1
37
25
Ascl1
20
15
H3
kDa

Supplementary Table S1: List of primers for analysis of mouse gene expression by qRT-PCR

Gene	Left Primer	Right Primer
Ascl1	TTCTCCGGTCTCGTCCTACTC	CCAGTTGGTAAAGTCCAGCAG
Cdk1	CTTCGACATCCAAATATAGTCA	CCAGGAGGGATGGAGTCCAGGTACTT
Cdk2	GGGTCCATCAAGCTGGCAGA	TGCTCGGTACCACAGGGTCACC
EiF1α	TAAAGAGGATGGGCAGGAGT	CTCCTCACACCGTCAAAGCA
E2f1	TAGCCCTGGGAAGACCTCAT	CATCCGTGGCAATACTGCTT
Neurod1	CGCAGAAGGCAAGGTGTC	TTTGGTCATGTTTCCACTTCC
Skp2	CATGTGCTGTACCCGGAAAGA	ACTGTGCGCTTGCTCTCCC
Tubb3	ATTCTGGTGGACTTGGAACCT	ACTCTTTCCGCACGACATCT

Supplementary Table S2: List of plasmids used in this study

Mutation of Ascl1 lysines was performed by changing the cDNA sequence with the QuickChange II Mutagenesis kit (Agilent Technologies) following the manufacturer's instructions.

Recombinant gene	Species	Source
pCS2^{+}-wt_Ascl1	Human	Ali FR et al., 2014[1]
pCS2^{+}-N-term K>R_Ascl1	Human	This paper
pCS2^{+}-bHLH K>R_Ascl1	Human	This paper
pCS2^{+}-Full K>R_Ascl1	Human	This paper
pGL4.26-ΔM short-Luciferase	Mouse	Cloned for this paper based on Castro DS et al., 2006[2]
Renilla-TK-Luciferase		Promega (E2241)

References:

1 Ali, F. R. *et al.* The phosphorylation status of Ascl1 is a key determinant of neuronal differentiation and maturation in vivo and in vitro. *Development* **141**, 2216-2224, doi:10.1242/dev.106377 (2014).

2 Castro, D. S. *et al.* Proneural bHLH and Brn proteins coregulate a neurogenic program through cooperative binding to a conserved DNA motif. *Developmental cell* **11**, 831-844, doi:10.1016/j.devcel.2006.10.006 (2006).
